# Genome-wide association meta-analysis identifies five novel loci for age-related hearing impairment

**DOI:** 10.1038/s41598-019-51630-x

**Published:** 2019-10-23

**Authors:** Andries Paul Nagtegaal, Linda Broer, Nuno R. Zilhao, Johanna Jakobsdottir, Charles E. Bishop, Marco Brumat, Mark W. Christiansen, Massimiliano Cocca, Yan Gao, Nancy L. Heard-Costa, Daniel S. Evans, Nathan Pankratz, Sheila R. Pratt, T. Ryan Price, Christopher Spankovich, Mary R. Stimson, Karen Valle, Dragana Vuckovic, Helena Wells, Gudny Eiriksdottir, Erik Fransen, Mohammad Arfan Ikram, Chuang-Ming Li, W. T. Longstreth, Claire Steves, Guy Van Camp, Adolfo Correa, Karen J. Cruickshanks, Paolo Gasparini, Giorgia Girotto, Robert C. Kaplan, Michael Nalls, John M. Schweinfurth, Sudha Seshadri, Nona Sotoodehnia, Gregory J. Tranah, André G. Uitterlinden, James G. Wilson, Vilmundur Gudnason, Howard J. Hoffman, Frances M. K. Williams, André Goedegebure

**Affiliations:** 1 Department of Otorhinolaryngology, Erasmus Medical Center, 3015 CE Rotterdam, The Netherlands; 2 Department of Internal Medicine, Erasm us Medical Center, 3015 CE Rotterdam, The Netherlands; 3 Icelandic Heart Association, Holtasmari 1, Kopavogur, IS-201 Iceland; 4 Department of Otolaryngology and Communicative Sciences, The University of Mississippi Medical Center, 2500 North State Street, Jackson, MS 39216 USA; 5 Department of Medicine, Surgery and Health Sciences, University of Trieste, Trieste, Italy; 6 Cardiovascular Health Research Unit, University of Washington, Seattle, WA 98195 USA; 7 Medical Genetics, Institute for Maternal and Child Health, IRCCS “Burlo Garofolo”, Trieste, Italy; 8 Department of Physiology and Biophysics, The University of Mississippi Medical Center, 2500 North State Street, Jackson, MS 39216 USA; 9 NHLBI Framingham Heart Study, Framingham, MA 01702 USA; 10 Department of Epidemiology and Biostatistics, University of California, San Francisco, CA 94158 USA; 11 Department of Laboratory Medicine and Pathology, University of Minnesota, Minneapolis, MN 55455 USA; 12 Department of Communication Science & Disorders, University of Pittsburgh, 6035 Forbes Tower, Pittsburgh, PA 15260 USA; 13 Laboratory of Neurogenetics, National Institute on Aging, Bethesda, MD 20892 USA; 14 Jackson Heart Study, 350 W. Woodrow Wilson Blvd, Suite 701, Jackson, MS 39213 USA; 15 Department of Twin Research and Genetic Epidemiology, King’s College London, London, UK; 16 Center for Medical Genetics, University of Antwerp, Prins Boudewijnlaan 43/6, BE-2650 Edegem, Antwerp Belgium; 17 Department of Epidemiology, Erasmus Medical Center, 3015 CE Rotterdam, The Netherlands; 18 Epidemiology and Statistics Program, Division of Scientific Programs, National Institute on Deafness and Other Communication Disorders (NIDCD) National Institutes of Health (NIH), Neuroscience Center Building, Suite 8300, 6001 Executive Blvd, Bethesda, MD 20892 USA; 19 Departments of Neurology and Epidemiology, University of Washington, Seattle, WA 98195 USA; 20 Departments of Ophthalmology and Visual Sciences and Population Health Sciences, University of Wisconsin, Madison, WI 53726 USA; 21 Department of Epidemiology and Population Health, Albert Einstein College of Medicine, Bronx, NY 10461 USA; 22 Data Tecnica International, Glen Echo, MD 20812 USA; 23 Glenn Biggs Institute for Alzheimer’s & Neurodegenerative Diseases, UT Health, San Antonio, 78229 TX USA

**Keywords:** Genome-wide association studies, Genetics research

## Abstract

Previous research has shown that genes play a substantial role in determining a person’s susceptibility to age-related hearing impairment. The existing studies on this subject have different results, which may be caused by difficulties in determining the phenotype or the limited number of participants involved. Here, we have gathered the largest sample to date (discovery n = 9,675; replication n = 10,963; validation n = 356,141), and examined phenotypes that represented low/mid and high frequency hearing loss on the pure tone audiogram. We identified 7 loci that were either replicated and/or validated, of which 5 loci are novel in hearing. Especially the *ILDR1* gene is a high profile candidate, as it contains our top SNP, is a known hearing loss gene, has been linked to age-related hearing impairment before, and in addition is preferentially expressed within hair cells of the inner ear. By verifying all previously published SNPs, we can present a paper that combines all new and existing findings to date, giving a complete overview of the genetic architecture of age-related hearing impairment. This is of importance as age-related hearing impairment is highly prevalent in our ageing society and represents a large socio-economic burden.

## Introduction

Hearing loss with age is highly prevalent and accounts for a large socio-economic burden^1,2^. Presbycusis or age-related hearing impairment (ARHI) may lead to loss of productivity at work^3^, social withdrawal^4^ and depression^5^. ARHI is associated with cognitive decline^6^ and dementia although the precise relationship between the two is debated^7^. ARHI typically affects hearing thresholds bilaterally which is most pronounced in the higher frequency range^8^, while the age of onset and rate of progression are variable. The cochlea plays a vital role in its pathophysiology and signs of cochlear degeneration are present in cases with ARHI^9,10^.

The etiology of ARHI is multifactorial and includes genetic factors, environmental factors, and their interaction^11^. Heritability estimates of ARHI vary depending on the precise phenotype studied but are substantial, ranging from 36–70%^12–15^. However, the genetic architecture of ARHI remains unclear and to date few genetic variants have been convincingly identified in humans. There are many known genetic variants associated with hearing loss of different types^16^, but few have been identified as underlying ARHI, which include TJP2^17^, MYO6^18^, and WFS1^19^.

Owing to the difficulty of obtaining human cochlea tissue, much research into ARHI has been performed in mice where genetic manipulation and biochemical studies are relatively easy to perform^20^. Several loci – named *Ahl* - have been associated with ARHI, resulting in the identification of one causative gene: cadherin 23^21^. Mutations in the human homologue of *Ahl* are implicated in Usher syndrome 1D and nonsyndromic autosomal recessive deafness DFNB12^22^. Evidence of their contribution to ARHI in humans is still lacking.

ARHI is not easily quantifiable. The gold standard measure of hearing, pure tone audiometry, provides an audiogram with hearings thresholds at multiple frequencies. Normal values for thresholds are dependent on age and sex and show a skewed distribution. In previous genome-wide association studies (GWAS) investigators have used a variety of traits, including different ways of deriving information from the pure tone audiogram: absolute thresholds^23^, pure tone averages^23^, principal components^23–26^, and Z-scores^27^. Health record ICD-9 diagnoses^28^ have also been used in absence of audiometry. This has led to associations reported between ARHI and variants in the following genes: *GRM7*^24,27^, *IQGAP2*^24^, *SIK3*^26^, *ISG20*, *TRIOBP*, *EYA4* and *ILDR1*^28^. There is a lack of consistency between these studies though, which is probably explained by relatively modest sample sizes, given the likely small effect sizes of the variants. However, suboptimal definition of the phenotype, large genetic heterogeneity and the study of isolated populations may also play a role in the failure to date to replicate many of the reported findings.

Here we present a large, 1000Gv3 imputed GWAS meta-analysis of ARHI using pure tone audiometry from multiple cohorts in Northern Europe and the USA, providing a total discovery cohort of 9,675 individuals. We were interested in exploring correlates of high and low/mid frequency hearing loss across samples of different ethnic backgrounds. Because previous work suggested an influence of ethnicity on ARHI, we performed the analyses both stratified by ancestry and in all ancestries combined.

## Results

### Discovery

**Figure 1:**
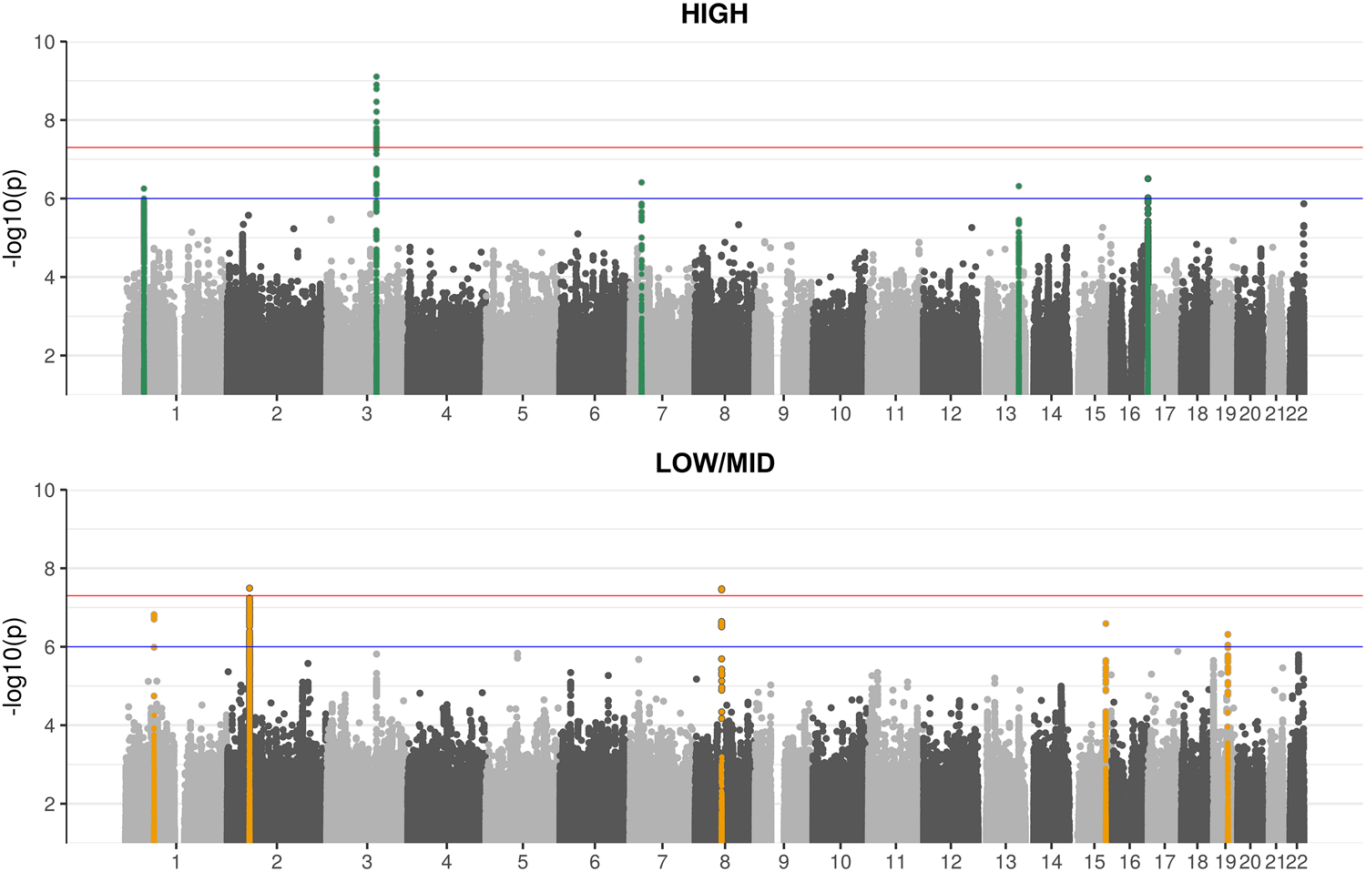
Manhattan plots for high and low/mid frequency hearing loss. The significant (P < 5*10^−8^; red horizontal line) and suggestive (P < 1*10^−6^; blue horizontal line) associations are coloured green for HIGH (high frequency hearing loss) and yellow for LOW/MID (low and mid frequency hearing loss). This colour scheme illustrates that high and low/mid frequency hearing loss have different genetic backgrounds. We found three genome-wide significant SNPs: one at chromosome 2 (rs6740893; LOW/MID phenotype), one at chromosome 3 (rs2332035; HIGH phenotype), and one at chromosomes 8 (rs9298078; LOW/MID phenotype).

Ten unique loci with suggestive or significant genome-wide P-values (Table 1 and Fig. 1) were identified. The HIGH phenotype yielded 5 associations (4 suggestive, 1 genome-wide significant), the LOW/MID phenotype also yielded 5 associations (3 suggestive, 2 significant). For the HIGH phenotype, the genome-wide significant SNP (rs2332035, P = 7.83*10^−10^) was located on 3q13.33 in the intron region within the *ILDR1* gene. For LOW/MID, the two genome-wide significant SNPs identified were rs6740893 (P = 3.22*10^−08^), located on 2p16.2 in the intron region within the *SPTBN1* gene, and rs9298078 (P = 3.36*10^−08^), located on 8q12.3 in an intronic non-coding RNA region. Interestingly, the HIGH and LOW/MID phenotypes did not show any overlap in associated signals, with all suggestive and significant loci being different between the two (Fig. 1). A Manhattan plot of all 4 phenotypes is included in Supplementary Fig. S1, while locus zoom plots of all genome-wide significant loci are available in Supplementary Fig. S2.

**Table 1:** Results of discovery, replication and validation for the HIGH and LOW/MID phenotypes.

Gene	SNP	chr	pos	EA	EAF	Discovery (n = 9,675)						Replication								Validation	
						HIGH			LOW/MID			European ancestry only				All ancestries				UK Biobank (n = 356,141)	
						beta	dir	P	beta	dir	P	beta	P	dir	N	beta	P	dir	N	beta	P
*IPP*	rs61784824	1	46211347	A	0.71	−0.083	−−−−−−	**5.59E-07**	−0.049	−−−−−−	1.94E-03	−0.089	**0.011**	?−	2,122	−0.018	0.244	?−+−	9,749	−0.032	**4.05E-08** ^*^
*CTH*	rs61776709	1	70994590	A	0.87	−0.083	−−−−−−	1.70E-04	−0.110	−−−−−−	**1.51E-07**	0.087	0.052	?++	2,158	0.012	0.520	?+++−	9,794	−0.007	4.00E-01
*SPTBN1*	rs6740893	2	54834380	A	0.23	0.062	++++++	3.39E-04	0.091	++++++	**3.22E-08** ^*^	0.002	0.954	−+	3,319	−0.019	0.221	−+−	10,955	0.029	**3.09E-06** ^*^
*ILDR1*	rs2332035	3	121715432	T	0.29	0.100	++++++	**7.83E-10** ^*^	0.070	++++++	4.77E-06	0.015	0.600	+++	3,283	−0.008	0.594	++++−	10,91	0.032	**1.47E-07** ^*^
*TRIL*	rs12112406	7	28937083	A	0.26	0.091	++++++	**3.88E-07**	0.059	++++++	4.99E-04	0.061	**0.032**	+++	3,283	−0.011	0.512	+++−	10,91	0.024	**7.21E-05** ^*^
*RP11-32K4.1*	rs9298078	8	64906619	T	0.05	0.144	++++++	2.99E-05	0.181	+−++++	**3.36E-08** ^*^	−0.011	0.867	+−+	3,319	−0.044	0.092	+−++−	10,955	−0.002	8.61E-01
*DOCK9*	rs1289319	13	99457063	T	0.60	−0.075	−−−−−−	**4.84E-07**	−0.048	−+−+−	7.68E-04	−0.001	0.974	+−+	3,283	−0.008	0.567	+−++−	10,91	0.001	8.59E-01
*ISG20*	rs56203268	15	89265679	T	0.83	0.086	++++++	2.51E-04	0.114	++++++	**2.57E-07**	−0.090	**0.034**	?−	2,158	0.005	0.808	?−+	9,794	0.037	**1.91E-07** ^*^
*SPIRE2*	rs6500458	16	89907205	A	0.42	−0.085	−+−-−−	**3.12E-07**	−0.036	−+−+−	2.21E-02	−0.067	**0.011**	−	3,283	−0.046	**0.002** ^*^	−−−−−	10,91	NA	NA
*FXYD5*	rs10403118	19	35677210	A	0.79	−0.046	−−−−+−	1.83E-02	−0.092	−−−−−−	**4.82E-07**	−0.066	**0.040**	−	3,319	−0.052	**0.007**	−+−	10,955	0.000	9.54E-01

HIGH: high frequency phenotype; LOW/MID: low and mid frequency phenotype; chr: chromosome; pos: position; EA: Effect allele; EAF: Effect allele frequency; dir: direction (for discovery cohorts: RS-II, RS-III, AGES, CHS, FHS, HABC; for replication cohorts: Antwerp, G-EAR, TwinsUK, JHS, HCHS/SOL). SNPs included are suggestively (P <1*10^−6^, listed in bold) or significantly (P < 5*10^−8^, indicated by an asterisk) associated in at least 1 phenotype. In replication and validation (UKB: UK Biobank): SNPs with nominally significant P-values (< 0.05) are listed in bold, SNPs significant after correcting for multiple testing (P = 0.005; 0.05/10 loci) are also indicated by an asterisk.

### Replication

Replication was performed for European ancestry only as well as all genetic ancestries combined (Table 1). Of the 5 SNPs found in the HIGH phenotype, 3 replicated at nominal significance levels (P < 0.05) in European ancestry only. One locus, rs6500458, was significantly replicated when combining replication samples from all ancestries (P = 0.002). For the LOW/MID phenotype 1 out of 5 SNPs replicated (rs10403118). One other SNP (rs56203268) was also significantly replicated, but the direction was inconsistent. Overall, we were able to replicate 4 SNPs lying on 4 separate chromosomes. Of note, the genome-wide significant SNP from the HIGH phenotype (rs2332035) was not formally replicated, but reached significance level in the meta-analysis of discovery and replication cohorts of European ancestry combined (P = 2.29*10^−8^; Supplementary Table S1).

### Clinical validation

The use of UK Biobank questionnaire data (as well as to identify other phenotypes linked to ARHI), was validated by exploring the phenotype genetic correlations using LDHub (Supplementary Table S2). A highly significant genetic correlation was identified between the questionnaire-based hearing loss phenotype in the UK Biobank and phenotype HIGH (rG = 0.751, P = 5.75*10^−7^). Of note, the second and third strongest trends from the LD Hub analyses were also related to hearing loss (‘Hearing aid user’ and ‘Hearing difficulty/problems with background noise’).

Ten independent loci were examined in UK Biobank using a phenotype derived from responses to questions about self-reported hearing loss. Of the 10 independent loci identified at the discovery phase, 5 loci (3 for HIGH and 2 for LOW/MID) achieved significance after Bonferroni correction (P < 0.005; Table 1). When considering both the formal replication with pure tone audiometry and the validation using UK Biobank, a total of 7 independent loci were identified for ARHI: 4 for high, and 3 for low/mid frequency hearing loss.

### Gene expression in the auditory system

A total of 20 genes were marked as candidate genes. In humans, 6 genes showed reproducible expression in the cochlea, 5 were marginally expressed, 3 were absent and data on 6 genes were not available (Table 2). In mice a majority of the genes (n = 15) were expressed within (inner or outer) hair cells. This number was somewhat lower in spiral ganglion cells: 10 out of 20 genes.

**Table 2:** Expression of annotated genes in the human and mouse inner ear and spiral ganglion cells.

Gene	Chr	Pheno-type	Human cochlea	MOUSE		
				Adult hair cells	Spiral ganglion cells	Differential expression in the inner ear?
*IPP*	1	H	P	P	P	no
*GPBP1L1*	1	H	P	P	A	no
*TMEM69*	1	H	P	P	A	no
*MAST2*	1	H	M	P	P	Utricle (FC 2.63; FDR 0.819), PN (FC 2.41; FDR 0.977)
*NASP*	1	H	P	P	P	no
*CTH*	1	L/M	n/a	A	A	—
*RNU6-1116P*	2	L/M	n/a	n/a	n/a	—
*SPTBN1*	2	L/M	P	P	P	no
*EML6*	2	L/M	M	A	P	no
*ILDR1*	3	H, L/M	n/a	P	A	HC (FC 7.65; FDR 1.33*10-4)
*TRIL*	7	H	n/a	P	P	SC (FC 2.5; FDR 0.243)
*RP11-32K4.1*	8	L/M	n/a	n/a	n/a	—
*SCUBE2*	11	L/M	P	P	P	PN (FC 2.06; FDR 0.927)
*DOCK9*	13	H	M	P	P	HC (FC 3.84; FDR 0.0273), PN (FC 2.24; FDR 0.0549)
*ISG20*	15	L/M	A	P	A	SC (FC 5.88; FDR 0.149); utricle (FC 4.76; FDR 0.353); PN (FC 4.63; FDR 0.514)
*VPS9D1*	16	H	A	P	P	no
*SPIRE2*	16	H	M	P	A	no
*SHC2*	19	L/M	n/a	P	A	HC (FC 6.69; FDR 2.07*10-3)
*ODF3L2*	19	L/M	A	P	n/a	HC (FC 2.40; FDR 0.0485); cochlea (FC 3.38; FDR 0.935); PN (FC 12.04; FDR 0.22)
*FXYD5*	19	L/M	M	A	P	—

Expression of the 20 annotated genes, as determined by proximity to the top SNP and MAGMA gene set analysis, in the human and mouse auditory system, by associated phenotype. For humans, only cochlear material was available. For mice, data on expression in adult (cochlear) hair cells and spiral ganglion cells (of the cochlear nerve) is listed, as well as differential expression within the inner ear. Abbreviations: H (high frequency phenotype), L/M (low/mid frequency phenotype). Expression in the human cochlea was classified as follows: n/a (not available), A (absent; < 1 fragments per kilobase of exon per million reads mapped (FPKM)), M (marginal; expression > 1 FPKM in 1 tissue sample), P (present; expression > 1 FPKM in more than 1 tissue sample). In mice, expression was investigated within the adult cochlea and spiral ganglion cells. Absent/present labels were designated according to >10.9 expression level or >=50% positive calls, respectively. Differential expression (defined as > 2-fold change) in the inner ear was noted between cell types (hair cells vs supporting cells), tissue types (cochlea vs utricle) and developmental stages (embryonic vs postnatal period). The accompanying fold changes (FC) and false discovery rates (FDR) are mentioned between brackets.

The following genes showed signs of differential expression in the inner ear: *MAST2* (utricle and postnatal period), *ILDR1* (hair cells), *TRIL* (supporting cells), *SCUBE2* (postnatal period), *DOCK9* (hair cells and postnatal period), *ISG20* (supporting cells, utricle, postnatal period), *SHC2* (hair cells) and *ODF3L2* (hair cells, cochlea, postnatal period). The greatest differential expression was noted for *ILDR1*, which is 7.65 times more expressed in hair cells than in supporting cells (FDR = 1.33*10^−4^).

### Genetic correlations

All 10 genetic variants identified as genome-wide suggestive or significant were either on different chromosomes or were far apart from each other (>10 Mb) and can therefore be considered independent loci. Only a very minor attenuation of signal was observed in the joint analysis (Supplementary Table S3).

A significant genetic correlation was identified between HIGH and LOW/MID phenotypes (rG = 0.69, P = 8*10^−5^) with SNP-based heritability (h2) more or less equal for the two: HIGH = 0.15 (P = 0.002); LOW/MID = 0.11 (P = 0.032). This implies that between 0.11–0.15 of the phenotypic variance can be explained by common SNPs with minor allele frequency > 0.05.

### Candidate gene approach

Results of the 7 candidate gene variants previously linked to ARHI are given in Table 3. No evidence for association between the two hearing phenotypes and the *GRM7* gene (rs11928865, rs779706 and rs779701) or the *IQGAP2* gene (rs457717) was found. We were unable to replicate the *SIK3* SNP (rs681524), which had been identified in the G-EAR consortium and TwinsUK. However, positive support was established for *ILDR1* (rs2877561 was highly significant in HIGH and LOW/MID), *ISG20* (rs4932196, highly significant in LOW/MID, and less so in HIGH) and *TRIOBP* (rs5756795, highly significant in LOW/MID) (Table 3). The direction of effect of these SNPs was the same in both studies. The SNP in *EYA4* (rs9493627) just failed to reach significance (P = 0.006 in LOW/MID).

**Table 3:** Lookup of previous GWAS findings.

SNP	chr	position	gene	EA	OA	EAF	HIGH			LOW/MID		
							beta	se	P	beta	se	P
rs11928865	3	7155702	*GRM7*	A	T	0.260	−0.0058	0.0170	0.7313	−0.0256	0.0160	0.1100
rs779706	3	7524042	*GRM7*	C	G	0.325	−0.0050	0.0180	0.7806	−0.0043	0.0171	0.7998
rs779701	3	7518772	*GRM7*	a	g	0.688	0.0038	0.0180	0.8322	0.0044	0.0169	0.7956
rs457717	5	75920972	*IQGAP2*	a	g	0.359	0.0079	0.0156	0.6151	0.0193	0.0148	0.1927
rs681524	11	116748314	*SIK3*	t	c	0.930	−0.0238	0.0307	0.4376	−0.0122	0.0289	0.6716
**rs4932196**	15	89253268	***ISG20***	t	c	0.803	0.0682	0.0192	**0.0004**	0.0841	0.0181	**3.44E-06**
**rs5756795**	22	38122122	***TRIOBP***	t	c	0.556	−0.0228	0.0148	0.1235	−0.0662	0.0141	**2.61E-06**
**rs2877561**	3	121712051	***ILDR1***	a	c	0.288	0.0986	0.0162	**1.26E-09**	0.0676	0.0155	**1.29E-05**
rs9493627	6	133789728	*EYA4*	a	g	0.338	0.0173	0.0155	0.2650	0.0402	0.0146	0.0060

All significant SNPs from previous GWA studies on ARHI were included. SNPs in *ILDR1*, *ISG20* and *TRIOBP* showed significant replication in our discovery cohort (P < 0.0056, Bonferroni corrected, in bold). *EYA4* was just above significance level. For *GRM7*, the phenotype consisted of Z-scores of frequencies 2, 4 and 8 kHz, normalized to means derived from the ISO standard^27^. Phenotyping for *IQGAP2* and *SIK3* was done through principal components analysis of audiometry^24,26^, while *EYA4*, *ILDR1*, *ISG20* and *TRIOBP* were found by using ICD-9 diagnoses of hearing loss^65^.

## Discussion

This study is the largest GWAS meta-analysis of ARHI to date and identified 7 associated loci, of which 5 are novel (*FXYD5*, *IPP*, *SPIRE2*, *SPTBN1* and *TRIL*) and 2 have been previously related to hearing loss (*ILDR1*, *ISG20*). Suggestive and significant associations showed no overlap between the low/mid and high frequency hearing loss phenotypes, indicating different pathophysiological mechanisms. In addition, we have confirmed some of the SNPs previously reported as related to ARHI (*ILDR1*, *ISG20* and *TRIOBP*), while others were not replicated (*EYA4*, *GRM7*, *IQGAP2* and *SIK3*). Our study has again shown that ARHI is highly polygenic as many genes - each with small effect - contribute to the estimated heritability of 36–70%^12–15^. In this study we identify a SNP-based heritability of 11–15%. This is in line with the observation that twin and family studies produce higher heritability estimates of common complex traits because GWAS estimates only the contribution made by common variants while other heritable variation (such as indels and CNVs) are largely ignored^29^.

At the outset, we chose to include only those cohorts having collected pure tone audiograms, as this is the gold standard measure of hearing ability and provides the best opportunity to define sub-phenotypes by which to interrogate putative pathologic pathways. Although ARHI initially impairs high frequency hearing, a correlate for low/mid frequency hearing loss was also included. Indeed, we found genetic variants associated with both HIGH and LOW/MID phenotypes and consistent with current understanding of cochlea pathology the associated variants were mutually exclusive.

The present study has a number of limitations. As our data are cross-sectional, we cannot exclude a contribution from other causes of hearing loss besides ARHI. However, congenital forms and other cochlea diseases will probably represent only a very small heterogeneous subset of the total number of participants. Second, replication was performed in a mixed ethnicity sample including European, African American and Hispanic heritage. We were unable to replicate most of our 10 discovery hits in non-Europeans, most likely due to a lack of power owing to the small sample size and maybe a different genetic architecture of ARHI. Indeed prevalence of ARHI differs by ethnicity, with African Americans having lower rates compared to non-Hispanic whites and Hispanics^30,31^. The prevalence of ARHI in Hispanics varies between different backgrounds^32^, but on average appears to be similar to non-Hispanic whites^33^.

The UK Biobank was used to validate our results based on responses to questions regarding hearing loss. Previous work on this dataset had examined the speech-in-noise phenotype and found it unreliable. A significant correlation was found between our HIGH phenotype and self-reported hearing loss in the UK Biobank, indicating that these responses may be a useful alternative to pure tone audiometry, in line with literature^34^. The Rotterdam study provides similar reassurance, where a high correlation between self-reported hearing loss and pure tone audiometry has been demonstrated (A.P.N., A.G., unpublished data). Despite different approaches to phenotyping, the UK Biobank questionnaire responses are useful in that they indicate the genetic variants responsible for pure tone audiometry changes are relevant to individuals’ clinical symptoms, something that is increasingly important in publically funded research.

Data on gene expression in the auditory system can provide supportive evidence, although such data should be interpreted with caution. The effects of aging may be manifest through other mechanisms, for example via the circulation, and may have an important influence on hearing function. Almost all associated genes identified showed signs of expression within the human or mouse cochlea, spiral ganglion cells or both. Of specific interest is the differential expression because most genes involved in hearing loss to date have been shown to be overexpressed within cochlear hair cells^35^.

The most highly associated SNP in our meta-analysis lies on chromosome 3 in close proximity to two genes: *SLC15A2* and *ILDR1*. The *SLC15A2* gene was initially considered a candidate gene for the nonsyndromic hearing loss locus, and was designated DFNB42^36^. However, sequencing of the *SLC15A2* gene yielded no causal variants. Six years later, the Ig-like domain containing receptor 1 (*ILDR1*) gene was identified as the causal gene^37^. Several mutations within the *ILDR1* gene have been associated with autosomal recessive nonsyndromic hearing loss to variable extent^37–41^. In addition, this gene has been linked previously to ARHI through a candidate gene approach^28^. The *ILDR1* protein mediates the recruitment of tricellulin to tight tricellular junctions, which plays a crucial role in the epithelial barrier function. *ILDR1* knock-out mice initially show normal development of inner and outer hair cells and the organ of Corti^42^. At 2 weeks of age however, outer hair cells begin to degenerate at the basal turn of the cochlea, corresponding to the higher frequencies. Later, this progresses to outer hair cells of the lower frequencies as well, and hearing function is severely diminished at 3 weeks of age. This process describes an accelerated version of the biology of ARHI in humans.

*SPTBN1*, encoding the spectrin beta, non-erythrocytic 1 protein, also known as βII spectrin, contains the top SNP of one of the low/mid frequency hearing loss loci. Spectrins are a major component of the cell membrane cytoskeleton and are located in hair and supporting cells of the cochlea^43^. As the cytoskeleton has a close relationship with outer hair cell electromotility, namely a shortening and elongation of the cell in response to sound stimuli, this may provide a mechanism through which *SPTBN1* contributes to hearing loss.

Cystathionine-γ-lyase (*CTH*) is involved in the formation of hydrogen sulfide and this gas has been demonstrated to regulate cochlear blood flow and has the ability to protect against noise-induced hearing loss^44^. *Odf3l2* has been listed as a candidate gene involved in mild hearing loss in a large-scale screen in mice^45^ and is differentially expressed in hair cells^35^. To our knowledge, no relationship has been established yet between hearing loss and any of the other annotated genes (*DOCK9*, *EML6*, *FXYD5*, *GPBP1L1*, *IPP*, *MAST2*, *NASP*, *SCUBE2*, *SHC2*, *SPIRE2*, *TMEM69*, *TRIL*, and *VPS9D1*). The precise mechanism through which they act on hearing function is yet unknown. Pathway analysis performed on our significantly associated variants was unrewarding.

Published genome-wide associations with ARHI are also of particular interest, as it confirmed associated variants in *ILDR1*, *ISG20* and *TRIOBP*, but calls into question the association of *GRM7*, which for a long time was considered proven because of the number of studies supporting its association. Considering differences that may arise through different phenotyping, the first study to identify the association between *GRM7* and ARHI also employed a Z-score approach to pure tone audiometry, but a slightly different method of calculation was used by normalizing according to the ISO standard^27^. It would seem unlikely that this difference in methodology should account for the contrasting results. Alternative explanations include a false positive finding based on smaller sample size or differences in the populations studied.

The *IQGAP2* gene, which also failed to replicate in our dataset was reported associated in one of the earlier GWA studies^24^. There are two major study differences: principal components of the pure tone audiogram were used instead of the Z-score method we employed. Second, the Finnish study was performed in an isolated population of the Saami using a small sample, so results may not be pertinent to the outbred Northern European sample of our work. Similarly, a study in a Han Chinese male sample investigating the relationship between *IQGAP2* and ARHI did not show any significant association^46^.

*SIK3* was identified in a GWAS meta-analysis of the G-EAR consortium and TwinsUK which included samples from outbred and inbred populations^26^. All samples except one contributed to the signal in the same direction and three samples were nominally significant. While the imputation quality of some of the samples was not high, this signal was confirmed in samples where whole genome sequencing was available. This finding was made in principal component 2, representing the slope of the audiogram, which corresponds to our HML phenotype. As the HML phenotype and some other studies that employed principal component 2^23–25^ did not produce the same results, the *SIK3* might also be a false positive finding, though it is expressed in mouse cochlea.

*ISG20* was replicated in our dataset, but the mechanism by which this gene affects hearing is unknown. It is barely expressed within the cochlea but is demonstrable in spiral ganglion cells, especially at later stages of development^47^. Interestingly, more interferon-related genes show signs of differential expression during late development and adulthood. Interferons are required for central nervous system homeostasis^48^, perhaps also in spiral ganglion cells.

*TRIOBP* (TRIO- and F-actin binding protein) is another gene that was replicated in our discovery cohort and is a well-established hearing loss gene, labelled as DFNB28^49^. The TRIOBP protein is localized in the rootlets of hair cell stereocilia and provides stability and rigidity. In knock-out mice, stereocilia develop normally but are easier to deflect and damage^50^. Initially thought to be responsible for profound, early onset hearing loss, mutations in *TRIOBP* have been recently shown to be also linked to late onset^28^ and more moderate hearing loss^51^, fitting the description of ARHI well.

To conclude, this study provides a large step forward in unravelling the genetic architecture of ARHI. Future studies have to confirm the associated loci and elucidate the pathophysiological pathways in which they may lead to hearing loss.

## Methods

Six population-based studies from the CHARGE (Cohorts for Heart and Aging Research in Genomic Epidemiology) consortium^52^ that have collected pure tone audiometry were included. Participants from Age, Gene/Environment Susceptibility Study (AGES; n = 3,104), Cardiovascular Health Study (CHS; n = 327), Framingham Heart Study (FHS; n = 1,978), Health ABC (HABC; n = 1,174), and the Rotterdam study (RS cohorts II and III; n = 3,092) were combined to provide a discovery sample of n = 9,675. The replication samples had also obtained pure tone audiometry and included the Antwerp study (n = 1,161), the G-EAR consortium (n = 1,339), the Jackson Heart Study (JHS; n = 735), Hispanic Community Health Study/Study of Latinos (HCHS/SOL; n = 6,909) and TwinsUK (n = 819), leading up to a total available sample of n = 10,963. Findings were further validated in the UK Biobank sample (https://www.ukbiobank.ac.uk/) using responses to questions on self-reported hearing loss because the speech-in-noise measures were found to be unreliable. In every analysis, only males and females aged 45 years or older were included. All participants provided written informed consent. Ethics approval was obtained locally at each study site: the Icelandic National Bioethics Committee (AGES); the institutional review boards at the University of Pittsburgh, the Johns Hopkins School of Public Health, Wake Forest University Health Sciences and the University of California Davis (CHS); the Boston Medical Center Institutional Review Board (FHS); the institutional review boards at the University of Pittsburgh and the University of Tennessee, Memphis (HABC); the Medical Ethics Committee of the Erasmus Medical Center (RS); the Committee for Medical Ethics UZA-UAntwerp; the Institutional Review Board of IRCCS Burlo Garofolo (G-EAR); the institutional review boards at the San Diego State University, the University of Miami, the University of North Carolina, the University of Illinois and the Albert Einstein College of Medicine (HCHS/SOL); the Institutional Review Board of the University of Mississippi Medical Center (JHS); and the National Research Ethics service London-Westminster (TwinsUK). The Declaration of Helsinki was adhered to. A more detailed description of the discovery and replication cohorts is available in the supplementary information.

### Phenotype description

Pure tone audiometry (air conduction thresholds at frequencies 0.5, 1, 2, 4 and 8 kHz) was collected on participants in all studies. We elected to examine hearing as a quantitative trait, rather than arbitrarily assign participants to case or control status. For each participant, the results of the better ear were used, defined as having the lowest threshold averaged over 0.5, 1, 2 and 4 kHz; when these were equal across ears we took the ear with the lowest threshold averaged over 4 and 8 kHz; if these were similar, the ear with the lowest threshold averaged over 0.5, 1 and 2 kHz was chosen; and if these too were similar, the results of the left ear were selected.

We found that ISO 7029 mean and standard deviation of hearing^53^ did not summarise our data well so decided that a standardised approach would allow each cohort to use its own data to generate means and standard deviations. Thus, each cohort provided its own reference panel and the effect of variations between audiometry centers was hereby minimised. Definition and calculation of the phenotypes was established before commencing the genetic analysis and not altered *post hoc*. Age-related hearing impairment mainly affects thresholds of the higher frequencies (Supplementary Fig. S3), showing a marked increase in hearing thresholds above 2 kHz (Supplementary Table S4, data from the Rotterdam Study). This has led to the decision to define a phenotype for high frequency hearing loss above 2 kHz and a second, separate one, that reflects hearing loss at the remaining low and mid frequencies. Age- and sex-adjusted weighted Z-scores of combinations of air conduction thresholds at frequencies 0.5 to 8 kHz were generated and mean thresholds of the following combinations were used to define:

- HIGH: high frequency hearing loss: 4 and 8 kHz
- LOW/MID: low and mid frequency hearing loss: 0.5, 1 and 2 kHz

Z-scores were generated by plotting mean thresholds against age using a linear regression model, with males and females considered separately. In contrast to the ISO 7029 standard, a quadratic function did not result in lower residuals so the simplest function, namely a linear function, was chosen. Data distributions were positively skewed, so a separate standard deviation was calculated for participants above and below the regression line. The residuals were each divided by the appropriate standard deviation, resulting in the phenotype. A positive value thus reflects a larger hearing loss than expected for age. An example R code to calculate the phenotype is provided in the supplementary information. For clarity purposes, the analysis on two additional phenotypes was excluded from the main text but made available in the supplementary information.

### Genotyping by center

Details of genotyping platforms, quality control and imputation to 1000Gv3 are available in Supplementary Table S5. Linear regression analysis was performed by each cohort adjusting only for cohort specific covariates (e.g. principal components, center, relatedness), because age and gender were already incorporated within the phenotype definition. Cohort summary statistics underwent quality control using EasyQC based on the standard protocol^54^, using a 1% minor allele frequency threshold.

### Discovery

Inverse variance-based meta-analysis of summary statistics was performed in METAL^55^. The most highly associated SNPs were considered for replication if they were genome-wide significant (P < 5*10^−8^) or suggestively associated (P < 1*10^−6^). Comparison of findings between the phenotypes was considered to be informative of the genetic relationship between specific characteristics of the audiogram (low/mid versus high frequency hearing loss).

### Replication

The same phenotype definition was used in five independent studies in which we attempted to replicate the most highly associated SNPs: the Antwerp study, the G-EAR consortium, JHS, HCHS/SOL and TwinsUK (details on genotyping are listed in Supplementary Table S5). They provided a total of 10,963 participants. These cohorts were of European ancestry except for JHS (African American ancestry; n = 735) and HCHS/SOL (Hispanic ancestry; n = 6,909). A combined meta-analysis of discovery and replication was performed both ancestry-specific, as well as for all ancestries combined (Supplementary Table S1).

All suggestive and significant loci from the discovery meta-analysis (P < 1*10^−6^) were considered for replication. Inverse variance-based meta-analysis of summary statistics was performed in METAL.

### Clinical validation

We sought to validate our findings further using UK Biobank. This national bioresource in the UK comprises ~500,000 participants aged 40–69 years registered with a general practitioner, and has been extensively described elsewhere^56^. Ethics approval was provided by the North West Multi-Center Research Ethics Committee (MREC). Participants were invited to local examination centers and underwent a battery of clinical tests and completed online questionnaires including questions pertaining to hearing ability. We elected at the outset to use responses to hearing ability questions to see if we could validate the genetic findings using a subjective measure that reflects clinical hearing disability. Participants had been asked the question ‘Do you have any difficulty with your hearing?’ Cases were defined as responding ‘Yes’ or ‘I am completely deaf’ while control status was assigned to those answering ‘No’. Participants with missing data and those aged below 45 years at the time of participation were excluded from the present study. GWAS was performed using Northern Europeans alone as defined by the response to the ethnicity question and principal component analysis.

PLINK2 logistic regression models were used to test for association adjusting for age, genetic sex (inferred from genotype data), UK Biobank PCs 1–10 and genotyping platform. Data were pre-processed by UK Biobank before release, and further QC was performed based on their recommended filters for excess relatedness (<10 putative 3^rd^ degree relatives in the kinship table), putative sex chromosome aneuploidy, heterozygosity and missing rates^57^. The final sample, selecting Northern Europeans as determined genetically, comprised n = 98,816 cases and n = 257,325 controls. Since 10 independent SNPs were investigated results were considered significant if P < 0.005, with Bonferroni correction.

### Gene expression in the auditory system

All suggestive and significant SNPs from the discovery cohort were included. Given that eQTL data were not available for the cochlea, candidate genes were selected in two ways. First, a candidate gene was assigned to each locus based on proximity to the top SNP. Second, in FUMA-GWAS^58^, a MAGMA gene-based test was used to link associated SNPs to genes and gene-phenotype association was examined using a burden test^59^. Genes with P < 2.8*10^−6^ were considered significantly associated with the phenotype of interest.

The cochlea is a distinct, highly differentiated organ with its own specific gene expression pattern. Human cochlea material is difficult to obtain so expression data are relatively lacking. We assessed a database, the only existing one as far as we are aware, derived from three human cochlear specimens^60^. Threshold for expression was set at FPKM > 1 (fragments per kilobase of exon per million reads mapped). Interpretation was performed in a qualitative manner, assigning results to one of four categories: not available (data on specific gene not available or did not pass quality control); absent (expression < 1 FPKM); marginal (expression > 1 FPKM in only one sample); or present (expression > 1 FPKM in more than one sample).

The Shared Inner Ear Laboratory Database (SHIELD) was also consulted, as it contains a wide variety of information on gene expression in the mouse auditory system that is unavailable in the human variant^61^. We looked at whether the candidate genes were expressed in adult cochlear inner and outer hair cells, at P25–P30^62^, and spiral ganglion cells of the cochlear nerve, at P15^47^. Expression was designated “present” when, respectively, levels exceeded 10.9 or >=50% of detection calls were positive. In addition, differential expression in the inner ear, defined as >2-fold change, was noted, looking at: cell type (hair cells vs supporting cells); tissue source (cochlea vs utricle); and developmental stage (embryonic vs postnatal period)^35^. False discovery rate was used to assess statistical significance.

### Genetic correlations

Independence of all suggestively and significantly associated variants was examined. Genome-wide Complex Trait Analysis (GCTA) was used to identify secondary signals employing the cojo-slct function^63^. A combined dataset of the Rotterdam II and III cohorts was used as genetic background.

LD score regression was employed to investigate the genetic correlation between the phenotypes, as well as to examine the genetic correlation between our phenotypes and publically available data on LD Hub^64^. Only traits investigated in European ancestry samples were considered. If a trait was included multiple times, the largest dataset was considered. Traits for which no estimates could be obtained were excluded. This approach resulted in 518 traits being investigated, including 339 unpublished traits from the UK Biobank, of which three were hearing-related phenotypes. Significance was set at Bonferroni adjusted P < 9.65*10^−5^.

### Candidate gene approach

We were interested to determine the strength of evidence in this dataset supporting SNPs previously identified as associated with ARHI. We used the discovery cohort to examine 9 genetic variants in 7 genes that have been previously associated with ARHI using agnostic study methods and replicated, or identified through GWAS meta-analysis: *GRM7*^27^; *IQGAP2*^24^; *SIK3*^26^; *EYA4*, *ILDR1*, *ISG20*, and *TRIOBP*^28^. Significance level was Bonferroni adjusted for 9 SNPs and set at P = 0.0056.

## Supplementary information

Supplementary information

Supplementary Table S2

Supplementary Table S5

## Data Availability

The datasets generated during and/or analysed during the current study are available from the corresponding author on reasonable request.
