# Supplementary material for: Genome-wide association meta-analysis identifies five novel loci for age-related hearing impairment
Source: Sci Rep. 2019 Oct 23;9:15192. doi: 10.1038/s41598-019-51630-x (PMC6811684; doi:10.1038/s41598-019-51630-x)
Supplement: Supplementary file 1 — Supplementary information [file 41598_2019_51630_MOESM1_ESM.docx]

SUPPLEMENTARY INFORMATION

Genome-wide association meta-analysis identifies five novel loci for age-related hearing impairment

Andries Paul Nagtegaal, Linda Broer, Nuno R. Zilhao, Johanna Jakobsdottir, Charles E. Bishop, Marco Brumat, Mark W. Christiansen, Massimiliano Cocca, Yan Gao, Nancy L. Heard-Costa, Daniel S. Evans, Nathan Pankratz, Sheila R. Pratt, T. Ryan Price, Christopher Spankovich, Mary R. Stimson, Karen Valle, Dragana Vuckovic, Helena Wells, Gudny Eiriksdottir, Erik Fransen, Mohammad Arfan Ikram, Chuang-Ming Li, WT Longstreth Jr, Claire Steves, Guy Van Camp, Adolfo Correa, Karen J. Cruickshanks, Paolo Gasparini, Giorgia Girotto, Robert C. Kaplan, Michael Nalls, John M. Schweinfurth, Sudha Seshadri, Nona Sotoodehnia, Gregory J. Tranah, André G. Uitterlinden, James G. Wilson, Vilmundur Gudnason, Howard J. Hoffman, Frances M.K. Williams, André Goedegebure

Two additional phenotypes were investigated, which were calculated in the same manner as the HIGH and LOW/MID phenotypes presented in the main text:

- HML: high (4 & 8 kHz) minus low/mid (0.5, 1 & 2 kHz) frequencies, reflecting the slope of the audiogram

- Frequencies used in the World Health Organization (WHO) classification of hearing loss: .5, 1, 2 and 4 kHz

In the meta-analysis of the discovery cohort, the WHO phenotype yielded 5 associations (2 suggestive, 3 significant), and the HML phenotype produced 3 associations (all suggestive). All 5 suggestive and significant loci from the WHO and 1 from the HML phenotype were also identified in the HIGH and LOW/MID phenotypes. For these loci, the top SNP from the HIGH or LOW/MID phenotypes was used for replication and validation (main text). Neither of the two suggestive SNPs in the HML phenotype was replicated.

Highly significant genetic correlations were identified between the audiogram-derived phenotypes. Genetic correlations (rG) were highest for WHO with HIGH (rG=0.89, P = 5*10^-21^) and LOW/MID (rG=0.92, P = 2*10^-107^) phenotypes, as they share a large portion of the frequencies included. The HML phenotype only showed a significant correlation with HIGH (rG=0.81, P = 52*10^-14^), but not with LOW/MID (rG=0.13, P = 0.68) or WHO (rG=0.47, P = 0.10). The SNP-based heritability (h2) was as follows: WHO = 0.12 (P = 0.016); and HML = 0.12 (P = 0.018).


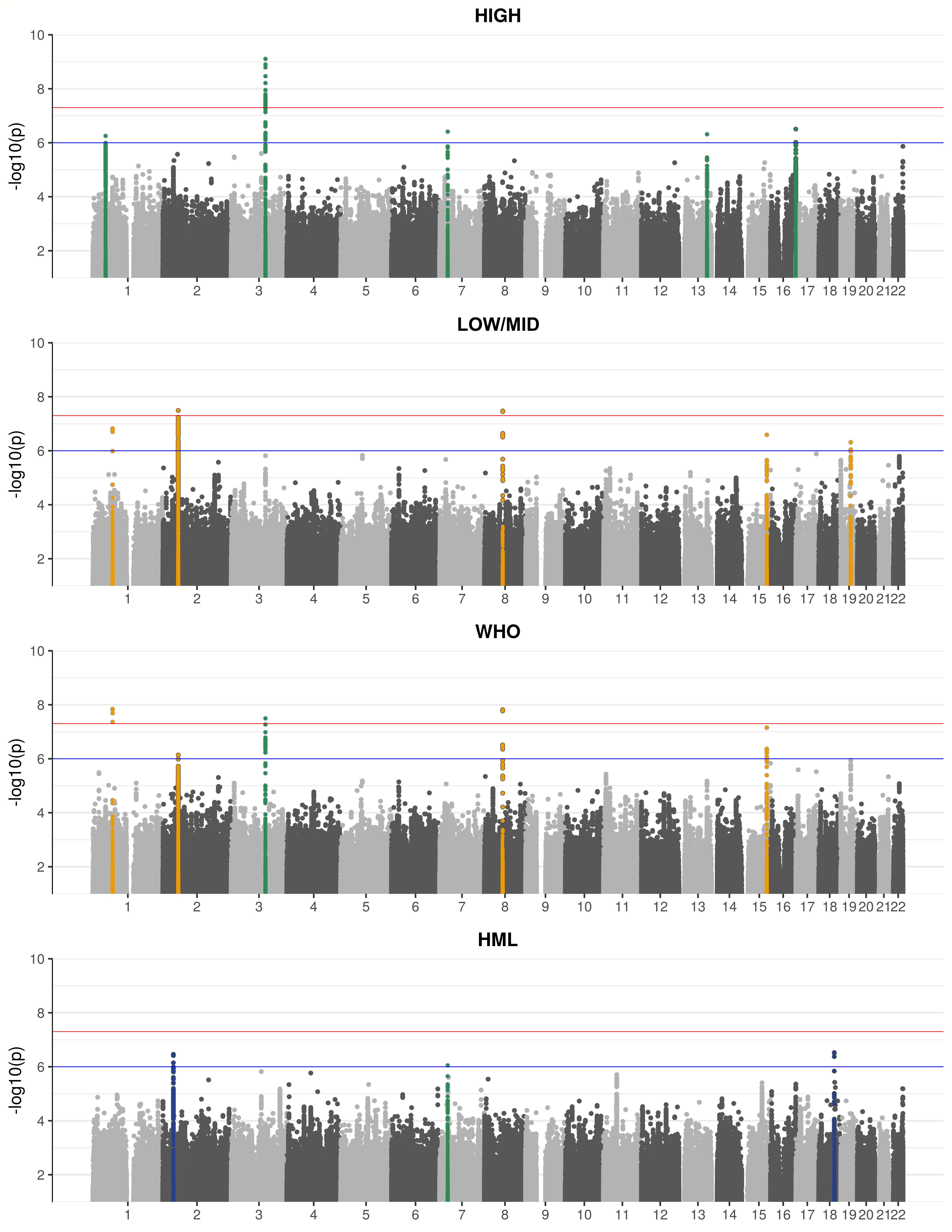


**Figure S1.**

Manhattan plots for all 4 phenotypes, showing the loci that were genome-wide significant (P < 5*10^-8^; red horizontal line) or suggestively associated (P < 1*10^-6^; blue horizontal line). The significant and suggestive associations are coloured green for HIGH (high frequency hearing loss), yellow for LOW/MID (low and mid frequency hearing loss), and blue for HML (high minus low frequency hearing loss). This colour scheme illustrates that all associations in WHO (phenotype using the WHO classification for hearing loss) were also seen in HIGH or LOW/MID. We found four genome-wide significant SNPs: one at chromosome 1 (rs61776709; WHO phenotype), one at chromosome 2 (rs6740893; LOW/MID and WHO phenotypes), one at chromosome 3 (rs2332035; HIGH and WHO phenotypes), and one at chromosomes 8 (rs9298078; LOW/MID and WHO phenotypes).

A) B)


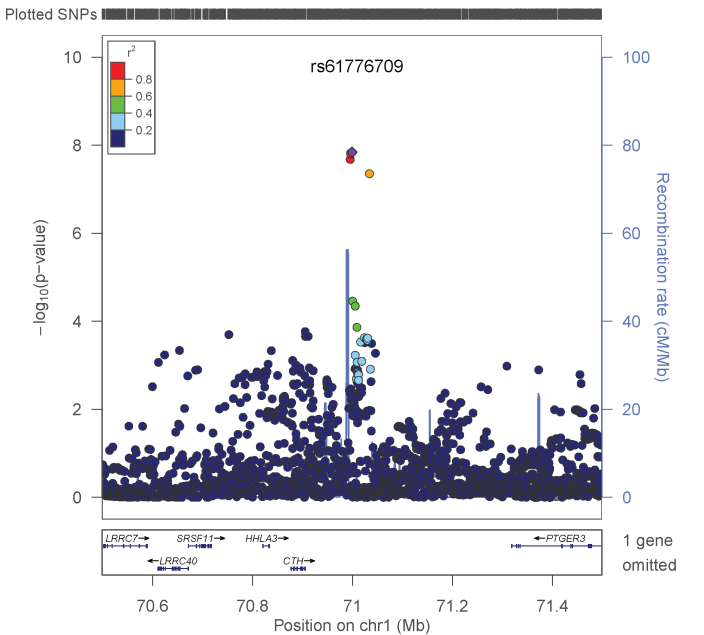

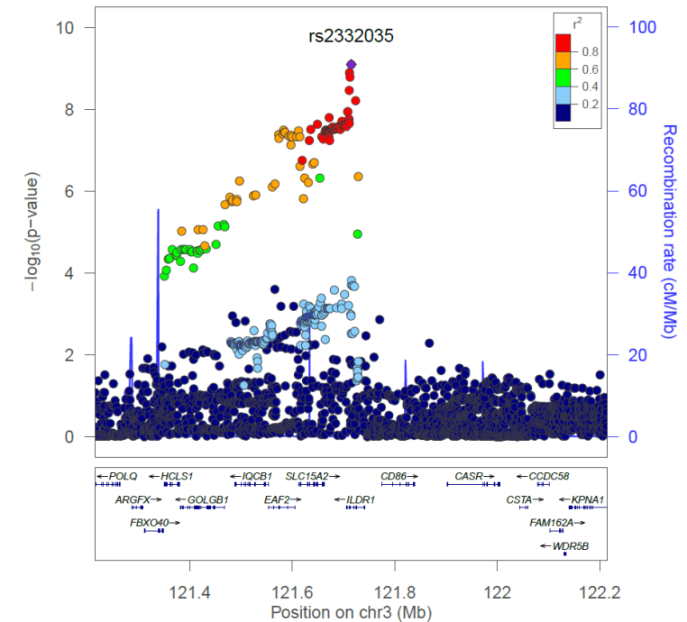


C)


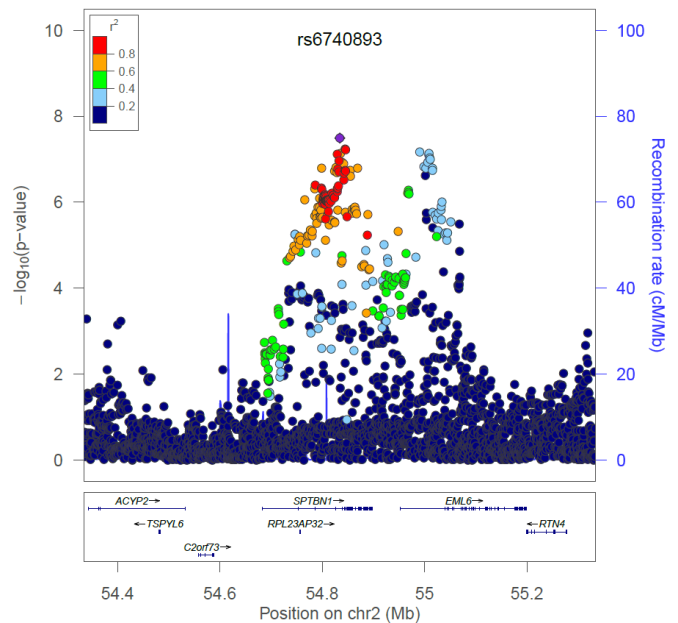

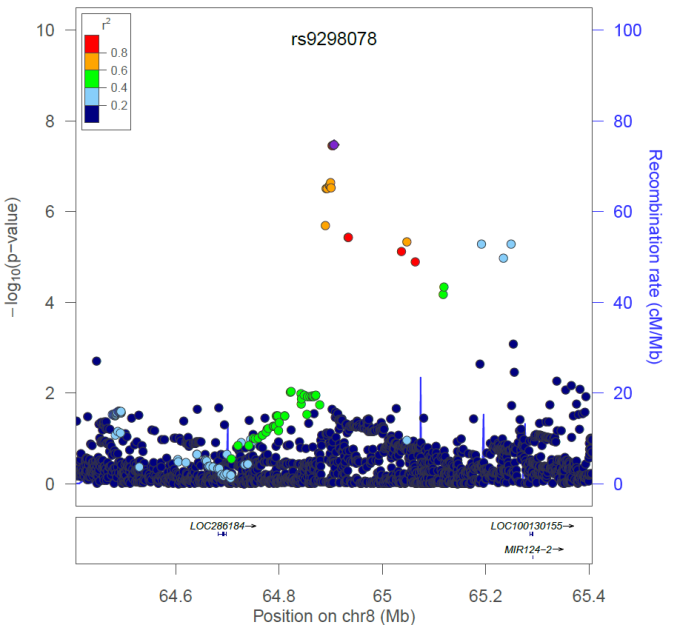


**Figure S2.**

Regional association plots of the four genome-wide significant SNPs (P < 5*10^-8^) for the (A) HIGH, (B) WHO, and (C) LOW/MID phenotypes. SNP rs2332035 is located in chromosome 3, within the ILDR1 gene; SNP rs61776709 is located intergenic, close to the *CTH* gene; SNP rs6740893 is located in chromosome 2 within the *SPTBN1* gene, and rs9298078 in chromosome 8 in a non-coding RNA intronic region. For each region, the top lead SNP is coloured dark purple and all independent significant SNPs are coloured red.


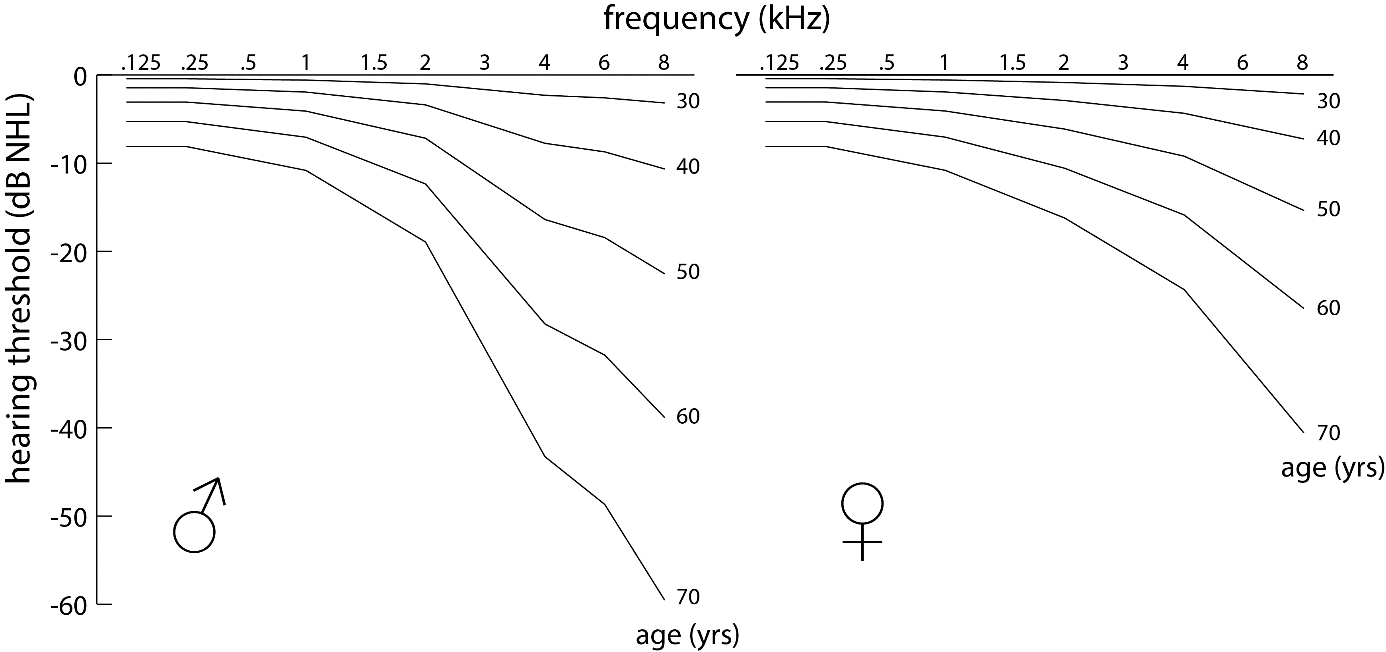


**Figure S3.**

Overview of age-related hearing impairment. Mean hearing thresholds for men and women, per decade^1^.

**Table S1: meta-analysis discovery + replication**

|  |  |  |  | Discovery | | | | Discovery+replication (EUR only) | | | | | | Discovery+replication (all) | | | | | |
| --- | --- | --- | --- | --- | --- | --- | --- | --- | --- | --- | --- | --- | --- | --- | --- | --- | --- | --- | --- |
| Pheno | SNP | EA | EAF | BETA | SE | PVAL | N | BETA | SE | PVAL | direction | I2 | N | BETA | SE | PVAL | direction | I2 | N |
| HIGH | rs61784824 | A | 0.71 | -0.083 | 0.017 | 5.59E-07 | 9,675 | -0.085 | 0.015 | 2.12E-08 | -?-- | 0 | 11,797 | -0.049 | 0.011 | 1.99E-05 | -?--+- | 73.5 | 19,424 |
| L/M | rs61776709 | A | 0.87 | -0.110 | 0.021 | 1.51E-07 | 9,675 | -0.075 | 0.019 | 7.80E-05 | -?++ | 88.9 | 11,833 | -0.043 | 0.014 | 2.21E-03 | -?+++- | 84.1 | 19,469 |
| WHO | rs61776709 | A | 0.87 | -0.121 | 0.021 | 1.48E-08 | 9,675 | -0.083 | 0.019 | 1.57E-05 | -?++ | 88.2 | 11,816 | -0.048 | 0.014 | 8.36E-04 | -?+++- | 84.5 | 19,460 |
| HML | rs2373109 | A | 0.51 | 0.075 | 0.015 | 3.46E-07 | 9,675 | 0.059 | 0.013 | 3.32E-06 | ++-- | 48.6 | 12,958 | 0.041 | 0.010 | 5.20E-05 | ++--++ | 56.6 | 20,578 |
| L/M | rs6740893 | A | 0.23 | 0.091 | 0.016 | 3.22E-08 | 9,675 | 0.071 | 0.015 | 8.64E-07 | +--+ | 61 | 12,994 | 0.033 | 0.011 | 3.29E-03 | +--+-- | 80.3 | 20,630 |
| HIGH | rs2332035 | T | 0.29 | 0.100 | 0.016 | 7.83E-10 | 9,675 | 0.078 | 0.014 | 2.29E-08 | ++++ | 58.2 | 12,958 | 0.041 | 0.011 | 1.59E-04 | +++++- | 81 | 20,585 |
| WHO | rs2332035 | T | 0.29 | 0.085 | 0.016 | 5.38E-08 | 9,675 | 0.065 | 0.014 | 2.40E-06 | ++-- | 65.5 | 12,977 | 0.030 | 0.011 | 5.99E-03 | ++--+- | 80.7 | 20,621 |
| HIGH | rs12112406 | A | 0.26 | 0.091 | 0.018 | 3.88E-07 | 9,675 | 0.083 | 0.015 | 5.00E-08 | ++++ | 0 | 12,958 | 0.035 | 0.012 | 3.73E-03 | ++++-- | 82.2 | 20,585 |
| L/M | rs9298078 | T | 0.05 | 0.181 | 0.033 | 3.36E-08 | 9,675 | 0.140 | 0.029 | 1.46E-06 | ++-+ | 59.8 | 12,994 | 0.044 | 0.021 | 3.20E-02 | ++-++- | 83.4 | 20,630 |
| WHO | rs9298078 | T | 0.05 | 0.187 | 0.033 | 1.67E-08 | 9,675 | 0.142 | 0.029 | 1.34E-06 | +--+ | 66.8 | 12,977 | 0.053 | 0.021 | 1.03E-02 | +--++- | 81.9 | 20,621 |
| HIGH | rs1289319 | T | 0.60 | -0.075 | 0.015 | 4.84E-07 | 9,675 | -0.057 | 0.013 | 1.19E-05 | -+-+ | 70.9 | 12,958 | -0.040 | 0.010 | 1.04E-04 | -+-++- | 75.6 | 20,585 |
| L/M | rs56203268 | T | 0.83 | 0.114 | 0.022 | 2.57E-07 | 9,675 | 0.071 | 0.020 | 3.11E-04 | +?-- | 89.4 | 11,833 | 0.050 | 0.014 | 4.54E-04 | +?---+ | 81.7 | 19,469 |
| WHO | rs56203268 | T | 0.83 | 0.121 | 0.022 | 7.04E-08 | 9,675 | 0.075 | 0.020 | 1.71E-04 | +?-- | 90.2 | 11,816 | 0.055 | 0.014 | 1.13E-04 | +?---+ | 82.6 | 19,460 |
| HIGH | rs6500458 | A | 0.42 | -0.085 | 0.017 | 3.12E-07 | 9,675 | -0.080 | 0.014 | 1.28E-08 | ---- | 0 | 12,958 | -0.063 | 0.011 | 1.16E-08 | ------ | 6 | 20,585 |
| HML | rs12963741 | T | 0.13 | 0.109 | 0.021 | 2.98E-07 | 9,675 | 0.080 | 0.019 | 2.73E-05 | ++-- | 82.4 | 12,958 | 0.056 | 0.017 | 8.57E-04 | ++--+- | 80.6 | 20,578 |
| L/M | rs10403118 | A | 0.79 | -0.092 | 0.018 | 4.82E-07 | 9,675 | -0.086 | 0.016 | 6.71E-08 | ---- | 0 | 12,994 | -0.073 | 0.013 | 3.57E-08 | ----+- | 0 | 20,630 |

The meta-analysis was also performed with discovery and replication cohorts combined. In the second column, only participants with European ancestry were included, the third column depicts participants from all ancestries (including JHS and HCHS/SOL). Direction: total discovery cohort, Antwerp, G-EAR, TwinsUK, JHS, HCHS/SOL.

**Table S3: Test for independence (cojo analysis)**

| Phenotype | Chr | SNP | position | b | se | p | bJ | bJ_se | pJ |
| --- | --- | --- | --- | --- | --- | --- | --- | --- | --- |
| HIGH | 1 | rs61784824 | 46211347 | -0.08 | 0.02 | 5.59E-07 | -0.08 | 0.02 | 6.11E-07 |
| HIGH | 3 | rs2332035 | 121715432 | 0.10 | 0.02 | 7.83E-10 | 0.10 | 0.02 | 7.78E-10 |
| HIGH | 7 | rs12112406 | 28937083 | 0.09 | 0.02 | 3.88E-07 | 0.09 | 0.02 | 3.73E-07 |
| HIGH | 13 | rs1289319 | 99457063 | -0.08 | 0.01 | 4.84E-07 | -0.08 | 0.01 | 4.80E-07 |
| HIGH | 16 | rs6500458 | 89907205 | -0.08 | 0.02 | 3.12E-07 | -0.08 | 0.02 | 3.16E-07 |
| LOW/MID | 1 | rs61776709 | 70994590 | -0.110 | 0.021 | 1.51E-07 | -0.110 | 0.021 | 1.47E-07 |
| LOW/MID | 2 | rs6740893 | 54834380 | 0.091 | 0.016 | 3.22E-08 | 0.091 | 0.016 | 3.12E-08 |
| LOW/MID | 8 | rs9298078 | 64906619 | 0.181 | 0.033 | 3.36E-08 | 0.181 | 0.033 | 3.50E-08 |
| LOW/MID | 15 | rs56203268 | 89265679 | 0.114 | 0.022 | 2.57E-07 | 0.114 | 0.022 | 2.61E-07 |
| LOW/MID | 19 | rs10403118 | 35677210 | -0.092 | 0.018 | 4.82E-07 | -0.092 | 0.018 | 4.73E-07 |
| WHO | 1 | rs6686121 | 70998786 | -0.121 | 0.021 | 1.44E-08 | -0.121 | 0.021 | 1.62E-08 |
| WHO | 3 | rs71329249 | 121711346 | -0.088 | 0.016 | 3.20E-08 | -0.088 | 0.016 | 3.04E-08 |
| WHO | 8 | rs77005160 | 64905429 | -0.188 | 0.033 | 1.52E-08 | -0.188 | 0.033 | 1.60E-08 |
| WHO | 15 | rs56203268 | 89265679 | 0.121 | 0.022 | 7.04E-08 | 0.121 | 0.022 | 7.00E-08 |
| HML | 2 | rs2373109 | 37717796 | 0.075 | 0.015 | 3.46E-07 | 0.075 | 0.015 | 3.48E-07 |
| HML | 18 | rs12963741 | 55322011 | 0.109 | 0.021 | 2.98E-07 | 0.109 | 0.021 | 2.97E-07 |

GCTA cojo analysis including all significant and suggestive SNPs. Abbreviations: b/se/p: original beta/se/p-value; bJ/bJ_se/pJ: beta/se/p-value after conditioning for the other SNPs. Analysis performed per phenotype.

**Table S4: Average hearing thresholds per frequency**

|  | Males | | Females | |
| --- | --- | --- | --- | --- |
| Frequency (kHz) | Hearing threshold (dB) | SD (dB) | Hearing threshold (dB) | SD (dB) |
| .5 | 14.7 | 10.9 | 17.5 | 12.1 |
| 1 | 17.9 | 12.7 | 19.1 | 13.3 |
| 2 | 22.5 | 18.0 | 22.1 | 16.7 |
| 4 | 43.2 | 21.7 | 34.0 | 19.6 |
| 8 | 54.2 | 26.4 | 47.4 | 26.1 |

Average hearing thresholds (best ear) and standard deviation for men and women, per frequency, in the Rotterdam study.

**Study descriptives**

**Age, Gene/Environment Susceptibility** **Reykjavik Study: AGES-Reykjavik Study**

The Reykjavik Study cohort originally comprised a random sample of 30,795 men and women born in 1907-1935 and living in Reykjavik in 1967^2^. A total of 19,381 people attended, resulting in 71% recruitment rate. The study sample was divided into six groups by birth year and birth date within month. One group was designated for longitudinal follow up and was examined in all stages. One group was designated a control group and was not included in examinations until 1991. Other groups were invited to participate in specific stages of the study. Between 2002 and 2006, the AGES-Reykjavik study re-examined 5764 survivors of the original cohort who had participated before in the Reykjavik Study.

**Age-related hearing impairment (ARHI) study Antwerp**

A population-based sample was obtained through population registries made available by the local city councils. To make the population ethnically homogenous, we requested that at least three out of the four grandparents originated from the same region as the study subject. All responding subjects underwent clinical examination, otoscopy and completed a detailed questionnaire on medical history and exposure to environmental risk factors. A list of all questions and answers used in this study is available on request.

Strict exclusion criteria were applied to exclude persons having or having had a condition that possibly leads to hearing impairment. No phenotypic inclusion criteria were used for the sample collection. Subjects with ear diseases, possible monogenic forms of hearing impairment or other major pathologies with a possible influence on hearing, were excluded. The main goal was to study hearing impairment in healthy subjects and therefore persons with multiple hospitalizations were excluded. The complete list of exclusion criteria was previously reported^3^. In subjects passing the medical exclusion criteria, audiometric thresholds were determined for air conduction (0.25, 0.5, 1, 2, 3, 4, 6, 8 kHz) and bone conduction (0.5, 1, 2, 4 kHz) according to current clinical standards (ISO 8253). We excluded subjects with asymmetrical hearing loss (difference in air conduction threshold larger than 20 dB for at least 2 frequencies out of 0.5, 1 and 2 kHz). In case only one of the ears showed conductive hearing loss (air-bone gap of 15 dB or more at 0.5, 1 and 2 kHz), and in the absence of other exclusion criteria, the other ear could be included.

**Cardiovascular Health Study**

The Cardiovascular Health Study (CHS) is a population-based cohort study of risk factors for coronary heart disease and stroke in adults ≥65 years conducted across four field centers^4^. The original predominantly European ancestry cohort of 5,201 persons was recruited in 1989-1990 from random samples of the Medicare eligibility lists; subsequently, an additional predominantly African-American cohort of 687 persons was enrolled for a total sample of 5,888. Blood samples were drawn from all participants at their baseline examination and DNA was subsequently extracted from available samples. European ancestry participants were excluded from the GWAS study sample due to the presence at study baseline of coronary heart disease, congestive heart failure, peripheral vascular disease, valvular heart disease, stroke or transient ischemic attack or lack of available DNA. Genotyping was performed at the General Clinical Research Center’s Phenotyping/Genotyping Laboratory at Cedars-Sinai among CHS participants who consented to genetic testing and had DNA available using the Illumina 370CNV BeadChip system in 2007. Pure-tone hearing thresholds were obtained from the Pittsburgh, PA cohort of the Cardiac Health Study during the 11th year of the study (1999-2000). Pure-tone hearing testing was not conducted at the other study sites. Of the 560 participants tested, 547 had sufficiently complete hearing data. The racial composition of the group was 22.2% African American and 77.8% White of European decent. For the discovery analysis, data on European descent individuals were meta-analyzed with those of individuals from other cohorts. Mean age at testing was 80.6 years (70 – 96.7 years). The group was 58.1% female and 41.9% male, the mean years of formal education was 14.3 years (4 – 19 years), and income was relatively low with only 31.4% having an income of over $50,000 per year. General health was described as good to excellent by 91.1% of the group, and all were ambulatory. Only 25.1% of the participants reported having a hearing problem, with only 18.2% having a history of wearing hearing aids. A majority (89.9%) of the participants had worked in low-noise environments and 58.3% had a history of smoking tobacco. CHS was approved by institutional review committees at each field center and individuals in the present analysis had available DNA and gave informed consent including consent to use of genetic information for the study of cardiovascular disease.

**Framingham Heart Study**

The Framingham Heart Study is a prospective longitudinal investigation of the development of atherosclerosis and its clinical sequelae. Study participants were recruited at three time periods. The study was initiated in 1948-50 with the recruitment of 5209 individuals ages 28-62 (including some spouse pairs, parent-offspring pairs and siblings) for the purpose of investigating the multiple factors involved in the development of cardiovascular disease^5^. This group, known as the Original Cohort, has been examined every two years with a total of thirty-two examinations to date. In 1971-1975, offspring of the Original Cohort and the offspring spouses were recruited to examine among other goals the familial components of cardiovascular disease and its risk factors^6^. In 2002-2005, the third generation (children of the Offspring and grandchildren of the Original Cohort) was recruited^7^. The Offspring Cohort totaled 5124 and the Third Generation totaled 4095 at recruitment and have been examined every 4 to 8 years. The Offspring Cohort now has 9 examinations completed and the Third Generation has 2 examinations completed.

Between 1973 and 1975, hearing examinations were conducted on 2293 members of the original cohort, and between 1995 and 1999, identical examinations were conducted on 2262 members of the offspring cohort. Standard pure-tone audiograms were obtained on all participants using environments and meeting American National Standards Institute standards.

**G-EAR**

Within the International consortium called G-EAR, we used, for the replication studies, 1307 individuals coming from four isolated cohorts: Carlantino (CARL), a village located in South Eastern Italy; Friuli Venezia Giulia (FVG) Genetic Park which consists of 6 geographically isolated villages (Resia, Sauris, Clauzetto, Erto-Casso, San Martino del Carso, Illegio) located within the Friuli Venezia Giulia region (North-East Italy), Silk Road (SR) rural communities (which consists of samples coming from Georgia, Azerbaijan, Tajikistan, Armenia, Turkmenistan and Uzbekistan) and Val Borbera (VBI) population, a geographically isolated valley within the Appennine Mountains of Piedmont (North-West Italy). All tests were performed using standard audiometers. Subjects underwent pure-tone audiometry, tympanogram, and acoustic reflex testing in both ears. Measurements were all obtained after any acoustically obstructing wax had been removed. A questionnaire to obtain sociodemographic information, as well as data on physical activity (i.e. job, sport, etc.), lifestyle (e.g. smoking, alcohol consumption, coffee intake, diet including taste and food preferences, etc), clinical examinations (e.g. psycho- logical, neurological, cardiological, etc), clinical chemistry including blood count and more than 20 parameters, drugs, diseases and other information regarding the health status (e.g. body mass index, bone density, blood pressure, etc) have been collected for each subject. Only subjects aged 18 or older were included in the analysis. Clear familial forms of severe hearing loss have been excluded from the study.

**Health, Aging, and Body Composition (HABC) Study**

The HABC Study is a NIA-sponsored cohort study of the factors that contribute to incident disability and the decline in function of healthier older persons, with a particular emphasis on changes in body composition in old age. Between March 1997 and July 1998, 3075 70-79 year-old community-dwelling adults (41% African-American) were recruited to participate in the Health ABC Study. Medicare beneficiary listings were used to recruit in metropolitan areas surrounding Pittsburgh, Pennsylvania, and Memphis, Tennessee. Eligibility criteria included having no difficulty walking one-quarter of a mile, climbing 10 steps, or performing activities of daily living (transferring, bathing, dressing, and eating); no history of active treatment for cancer in the prior 3 years; and no plans to move from the area within 3 years.

**The Hispanic Community Health Study (HCHS)/Study of Latinos (SOL)**

HCHS/SOL is a community based prospective cohort study of 16,415 self-identified Hispanic/Latino persons aged 18-74 years at screening from randomly selected households in four U.S. field centers (Chicago, IL; Miami, FL; Bronx, NY; San Diego, CA) with baseline examination (2008 to 2011) and yearly telephone follow-up assessment for at least three years. HCHS/SOL cohort includes participants who self-identified as having Hispanic/Latino background, the largest groups being Central American (n=1,732), Cuban (n=2,348), Dominican (n=1,473), Mexican (n=6,472), Puerto-Rican (n=2,728), and South American (n=1,072). The goals of the HCHS/SOL are to describe the prevalence of risk and protective factors for chronic conditions (e.g. cardiovascular disease (CVD), diabetes and pulmonary disease), and to quantify all-cause mortality, fatal and non-fatal CVD and pulmonary disease, and pulmonary disease exacerbation over time. The baseline clinical examination^8^ included comprehensive biological (e.g., anthropometrics, blood draw, oral glucose tolerance test, ankle brachial pressure index, electrocardiogram), behavioral (e.g. dietary intake assessed with two 24-hour recalls, physical activity assessment by accelerometer and self-report, overnight sleep exam for apneic events, tobacco and alcohol assessed by self-report), and socio-demographic (e.g., socioeconomic status, migration history) assessments. Hearing testing occurred in sound-treated booths. Pure-tone air (0.5, 1, 2, 3, 4, 6, 8 kHz) and bone conduction (0.5, 2, 4 kHz) thresholds were measured using clinical audiometers (Grason-Stadler, Inc., Madison, WI) equipped with TDH-50P and insert earphones (E-A-Rtone 3A, Cabot Safety Corp., Indianapolis, IN) which were calibrated annually according to ANSI standards (S3.6-2010). A modified Hughson-Westlake procedure was used according to the guidelines of the American Speech-Hearing-Language Association. Masking was applied as necessary.

**Jackson Heart Study**

The JHS is a large, population-based observational study evaluating the etiology of cardiovascular, renal, and respiratory diseases among African Americans residing in the three counties (Hinds, Madison, and Rankin) that make up the Jackson, Mississippi metropolitan area. Data and biologic materials have been collected from 5,306 participants, including a nested family cohort of 1,498 members of 264 families. The age at enrolment for the unrelated cohort was 35-84 years; the family cohort included related individuals ≥21 years old. Participants provided extensive medical and social history, had an array of physical and biochemical measurements and diagnostic procedures, and provided genomic DNA during a baseline examination (2000-2004) and two follow-up examinations (2005-2008 and 2009-2012). The study population is characterized by a high prevalence of diabetes, hypertension, obesity, and related disorders. Annual follow-up interviews and cohort surveillance are ongoing. Hearing Status in the Jackson Heart Study Cohort, was funded in 2007, with data collection starting in January 2008. The five-year study was focused on assessment of peripheral and central auditory function of JHS participants by way of pure-tone audiometry, distortion product otoacoustic emissions, tympanometry, speech-in-noise, and dichotic processing. Additionally, data were collected on vestibular dysfunction as well as tinnitus and hearing handicap. Given that the human auditory system is highly vascular and sensitive to micro-vascular insult, the goal of the ancillary study was to assess whether signs of peripheral or central auditory dysfunction could serve as a bio-marker for risk of cardiovascular disease.

**The Rotterdam Study**

The Rotterdam study is a prospective, population-based cohort study among inhabitants of Ommoord, a district of Rotterdam, The Netherlands. As of 2008, 14,926 subjects aged 45 years or over comprise the cohort. Since 2016, it is being expanded by persons aged 40 years and over. The Rotterdam study targets cardiovascular, endocrine, hepatic, neurological, ophthalmic, psychiatric, dermatological, otolaryngological, locomotor, and respiratory diseases. The participants were all examined in some detail at baseline. They were interviewed at home (2 h) and then had an extensive set of examinations (a total of 5 h) in a specially built research facility in the center of the district. Written informed consent was obtained from all participants and the Medical Ethics Committee of the Erasmus Medical Center, Rotterdam, approved the study.

**TwinsUK**

TwinsUK is the only adult twin registry in the UK, comprising of over 12,000 healthy twin volunteers aged 16-98^9^. Collection of data and biologic materials commenced in 1992 and is ongoing. Twins have completed detailed health and lifestyle questionnaires, and attended clinical evaluations. The pure tone audiometry data was collected on a subset of the cohort (N=1242) between April 2010 and November 2012. Participants were recruited with an aim to study aging in females. An air-conduction pure-tone audiogram was conducted by trained personnel using a Madsen XETA audiometer including TDH39 headphones. All research was conducted according to the ethical standards as defined by the Helsinki declaration. Ethical approval for this study was obtained from the National Research Ethics service London-Westminster (REC reference number: 07/H0802/84). Written informed consent was obtained from all participants prior to study conduction. Participants were excluded from analysis based on missing data, male, age <45. There were 819 female participants aged >45 remaining for analysis.

**References**

1 International Organization for Standardization. Acoustics -- Statistical distribution of hearing thresholds related to age and gender. *ISO 7029:2000* (2000).

2 Harris, T. B. *et al.* Age, Gene/Environment Susceptibility-Reykjavik Study: multidisciplinary applied phenomics. *Am J Epidemiol* **165**, 1076-1087 (2007).

3 Van Eyken, E. *et al.* KCNQ4: A gene for age-related hearing impairment? *Hum. Mutat.* **27**, 1007-1016 (2006).

4 Fried, L. P. *et al.* The Cardiovascular Health Study: design and rationale. *Ann Epidemiol* **1**, 263-276 (1991).

5 Dawber, T. R., Kannel, W. B. & Lyell, L. P. An approach to longitudinal studies in a community: the Framingham Study. *Ann N Y Acad Sci* **107**, 539-556 (1963).

6 Feinleib, M., Kannel, W. B., Garrison, R. J., McNamara, P. M. & Castelli, W. P. The Framingham Offspring Study. Design and preliminary data. *Prev Med* **4**, 518-525 (1975).

7 Splansky, G. L. *et al.* The Third Generation Cohort of the National Heart, Lung, and Blood Institute's Framingham Heart Study: design, recruitment, and initial examination. *Am J Epidemiol* **165**, 1328-1335 (2007).

8 Sorlie, P. D. *et al.* Design and implementation of the Hispanic Community Health Study/Study of Latinos. *Ann Epidemiol* **20**, 629-641 (2010).

9 Moayyeri, A., Hammond, C. J., Valdes, A. M. & Spector, T. D. Cohort Profile: TwinsUK and healthy ageing twin study. *Int J Epidemiol* **42**, 76-85 (2013).

### Study specific acknowledgements

The **AGES-Reykjavik** study has been funded by NIH contract N01-AG012100, the NIA Intramural Research Program, an Intramural Research Program Award (ZIAEY000401) from the National Eye Institute, an award from the National Institute on Deafness and Other Communication Disorders (NIDCD) Division of Scientific Programs (IAA Y2-DC_1004-02), Hjartavernd (the Icelandic Heart Association), and the Althingi (the Icelandic Parliament). The study is approved by the Icelandic National Bioethics Committee, VSN: 00-063. The researchers are indebted to the participants for their willingness to participate in the study.

**Cardiovascular Health Study**: This CHS research was supported by NHLBI contracts HHSN268201200036C, HHSN268200800007C, HHSN268201800001C, N01HC55222, N01HC85079, N01HC85080, N01HC85081, N01HC85082, N01HC85083, N01HC85086; and NHLBI grants U01HL080295, R01HL087652, R01HL105756, R01HL085251, R01HL103612, R01HL120393, and U01HL130114 with additional contribution from the National Institute of Neurological Disorders and Stroke (NINDS). Additional support was provided through R01AG023629 from the National Institute on Aging (NIA). A full list of principal CHS investigators and institutions can be found at CHS-NHLBI.org. The provision of genotyping data was supported in part by the National Center for Advancing Translational Sciences, CTSI grant UL1TR001881, and the National Institute of Diabetes and Digestive and Kidney Disease Diabetes Research Center (DRC) grant DK063491 to the Southern California Diabetes Endocrinology Research Center. WT Longstreth, Jr has received NIH funding for co-investigator in CHS. The content is solely the responsibility of the authors and does not necessarily represent the official views of the National Institutes of Health.

The **Framingham Heart Study** is conducted and supported by the National Heart, Lung, and Blood Institute (NHLBI) in collaboration with Boston University (Contract No. N01-HC-25195 and HHSN268201500001). This manuscript was not prepared in collaboration with investigators of the Framingham Heart Study and does not necessarily reflect the opinions or views of the Framingham Heart Study, Boston University, or NHLBI. Funding for SHARe Affymetrix genotyping was provided by NHLBI Contract N02-HL- 64278. Nancy L. Heard-Costa is supported by NIH/NHLBI HHSN268201500001 (Ramachandran).

The **Health, Aging and Body Composition Study** is supported in part by the Intramural Research Program of the NIH, National Institute on Aging. This research was supported by NIA contracts N01AG62101, N01AG62103, N01AG62106 and NIA grant 1R03AG032498-01. The genome-wide association study was funded by NIA grant 1R01AG032098-01A1 to Wake Forest University Health Sciences and genotyping services were provided by the Center for Inherited Disease Research (CIDR). CIDR is fully funded through a federal contract from the National Institutes of Health to The Johns Hopkins University, contract number HHSN268200782096C. Mike A. Nalls’ participation is supported by a consulting contract between Data Tecnica International and the National Institute on Aging, NIH.

The **Hispanic Community Health Study (HCHS)/Study of Latinos (SOL)** was carried out as a collaborative study supported by contracts from the National Heart, Lung, and Blood Institute (NHLBI) to the University of North Carolina (N01-HC65233), University of Miami (N01-HC65234), Albert Einstein College of Medicine (N01-HC65235), Northwestern University (N01-HC65236), and San Diego State University (N01-HC65237). The following Institutes/Centers/Offices contribute to the HCHS/SOL through a transfer of funds to the NHLBI: National Center on Minority Health and Health Disparities, the National Institute of Deafness and Other Communications Disorders, the National Institute of Dental and Craniofacial Research, the National Institute of Diabetes and Digestive and Kidney Diseases, the National Institute of Neurological Disorders and Stroke, and the Office of Dietary Supplements. The Genetic Analysis Center at the University of Washington was supported by NHLBI and NIDCR contracts (HHSN268201300005C AM03 and MOD03). NIDCD funded the audiometric data collection of the Bronx site (Robert C. Kaplan) and the Hearing Coordinating Center (Karen J. Cruickshanks).

The **Jackson Heart Study (JHS)** is supported and conducted in collaboration with Jackson State University (HHSN268201800013I), Tougaloo College (HHSN268201800014I), the Mississippi State Department of Health (HHSN268201800015I/HHSN26800001) and the University of Mississippi Medical Center (HHSN268201800010I, HHSN268201800011I and HHSN268201800012I) contracts from the National Heart, Lung, and Blood Institute (NHLBI) and the National Institute for Minority Health and Health Disparities (NIMHD). The JHS Hearing Ancillary Study was funded by National Institute on Deafness and Other Communication Disorders (R01 DC008371). James G. Wilson is supported by U54GM115428 from the National Institute of General Medical Sciences. John M. Schweinfurth received an R01 NIDCD-funded grant to collect audiometric data. The authors also wish to thank the staffs and participants of the JHS. The views expressed in this manuscript are those of the authors and do not necessarily represent the views of the National Heart, Lung, and Blood Institute; the National Institutes of Health; or the U.S. Department of Health and Human Services.

The **Rotterdam Study** is funded by Erasmus Medical Center and Erasmus University, Rotterdam, Netherlands Organisation for the Health Research and Development (ZonMw), the Research Institute for Diseases in the Elderly (RIDE), the Ministry of Education, Culture and Science, the Ministry for Health, Welfare and Sports, the European Commission (DG XII), and the Municipality of Rotterdam. The authors are grateful to the study participants, the staff from the Rotterdam Study and the participating general practitioners and pharmacists. The generation and management of GWAS genotype data for the Rotterdam Study (RS I, RS II, RS III) was executed by the Human Genotyping Facility of the Genetic Laboratory of the Department of Internal Medicine, Erasmus MC, Rotterdam, The Netherlands. The GWAS datasets are supported by the Netherlands Organisation of Scientific Research NWO Investments (nr. 175.010.2005.011, 911-03-012), the Genetic Laboratory of the Department of Internal Medicine, Erasmus MC, the Research Institute for Diseases in the Elderly (014-93-015; RIDE2), the Netherlands Genomics Initiative (NGI)/Netherlands Organisation for Scientific Research (NWO) Netherlands Consortium for Healthy Aging (NCHA), project nr. 050-060-810. We thank Pascal Arp, Mila Jhamai, Marijn Verkerk, Lizbeth Herrera and Marjolein Peters, MSc, and Carolina Medina-Gomez, MSc, for their help in creating the GWAS database, and Karol Estrada, PhD, Yurii Aulchenko, PhD, and Carolina Medina-Gomez, MSc, for the creation and analysis of imputed data.

**TwinsUK:** The authors of this paper wish to express our appreciation to all study participants of the TwinsUK cohort. TwinsUK is funded by the Wellcome Trust, Medical Research Council, European Union, the National Institute for Health Research (NIHR)-funded BioResource, Clinical Research Facility and Biomedical Research Centre based at Guy’s and St Thomas’ NHS Foundation Trust in partnership with King’s College London. HRRW is funded by Action on Hearing Loss, CJS is funded under a grant from the Chronic Disease Research Foundation (CDRF). FMKW is supported by Arthritis Research UK grant number 20682.

**Example R function: how to calculate phenotype**

arhl.phenotype <- function(data,thresholds) {

# data is a data.frame with columns "id", "age", "sex" and the columns listed in thresholds (sex=1 for males, sex=2 for females)

# thresholds is a character vector of the column names of the hearing thresholds to use in z-score calculation

# usage:

# rownames(data) <- data$id (this is necessary to ensure phenotype can be matched to correct person)

# HIGH <- arhl.phenotype(data,c("khz4.better.ear","khz8.better.ear"))

# LOW <- arhl.phenotype(data,c("khz05.better.ear","khz1.better.ear","khz2.better.ear"))

# WHO <- arhl.phenotype(data,c("khz05.better.ear","khz1.better.ear","khz2.better.ear","khz4.better.ear"))

# data$HIGH <- HIGH

# data$LOW <- LOW

# data$WHO <- WHO

# prior to use make sure observations with missing data have been excluded from data

data$mean <- rowMeans(data[,thresholds])

male.resid <- resid(lm(mean~age,data=subset(data,sex==1)))

female.resid <- resid(lm(mean~age,data=subset(data,sex==2)))

male.mirrored.pos <- c(male.resid[male.resid>=0],-male.resid[male.resid>=0])

female.mirrored.pos <- c(female.resid[female.resid>=0],-female.resid[female.resid>=0])

male.mirrored.neg <- c(male.resid[male.resid<0],-male.resid[male.resid<0])

female.mirrored.neg <- c(female.resid[female.resid<0],-female.resid[female.resid<0])

male.sd.pos <- sd(male.mirrored.pos)

female.sd.pos <- sd(female.mirrored.pos)

male.sd.neg <- sd(male.mirrored.neg)

female.sd.neg <- sd(female.mirrored.neg)

male.z <- c(male.resid[male.resid>=0]/male.sd.pos,male.resid[male.resid<0]/male.sd.neg)

female.z <- c(female.resid[female.resid>=0]/female.sd.pos,female.resid[female.resid<0]/female.sd.neg)

z.score <- c(male.z,female.z)

z.score <- z.score[match(data$id,names(z.score))]

}
